# Self-Objectification and Cognitive Performance: A Systematic Review of the Literature

**DOI:** 10.3389/fpsyg.2020.00020

**Published:** 2020-01-28

**Authors:** Lara Winn, Randolph Cornelius

**Affiliations:** ^1^Psychology, Vassar College, Poughkeepsie, NY, United States; ^2^Vassar College, Poughkeepsie, NY, United States

**Keywords:** self-objectification, objectification theory, objectified body consciousness, body as object, cognitive performance, critical reasoning ability, cognitive functioning, cognitive load

## Abstract

Objectification theorists posit that exposure to sexually objectifying behavior, images, etc., leads women in particular to adopt an objectifying self-perspective. State self-objectification (SSO) (i.e., the internalization of the objectifying gaze) is theorized to usurp individuals' cognitive resources by diverting attention to their bodies. The objective of this paper is to systematically review the literature surrounding self-objectification and cognitive performance. Six databases retrieved 1,779 relevant articles. Studies were deemed eligible for inclusion if they (a) quantitatively investigated the relationship between SSO and cognitive performance using valid and reliable measures, (b) were published in a peer-reviewed journal between 1997 and 2019, inclusive, and (c) were available in English. Nine studies fulfilled all inclusion criteria. As the heterogeneity of the literature precluded meta-analysis, narrative synthesis was employed to review the results. While the quality of the studies was mixed, the results of our review support the contention that self-objectification impairs cognitive functioning. Appearance monitoring, actual-ideal self-discrepancies, negative self-conscious emotions, gender schema activation, and stereotype activation are evaluated as potential mechanisms behind the relationship between state self-objectification and cognitive performance, while chronic (trait) self-objectification is evaluated as a potential moderator.

Objectification theory posits that repeated experiences of sexual objectification socialize girls and women to adopt an evaluative third-person perspective on their bodies (McKinley and Hyde, 1996; Fredrickson and Roberts, 1997). Fredrickson and Roberts (1997) define sexual objectification as, “occur[ing] whenever a woman's body, body parts, or sexual functions are separated out from her person, reduced to the status of mere instruments, or regarded as if they were capable of representing her” (p. 175). Theorists suggest that girls and women are exposed to sexual objectification in three primary ways: (1) direct interpersonal experiences of objectification (e.g., unsolicited appearance commentary), (2) vicarious experiences of other women's objectification (e.g., overhearing men's appearance commentary about other women), and (3) objectified media representations of women (e.g., images in which women's bodies are fragmented) (Fredrickson and Roberts, 1997). All three forms of objectification are now commonly experienced by women on social networking sites such as Instagram, Facebook, and Twitter that have been developed since Fredrickson and Roberts' early work (see, for example, Bell et al., 2018; Ramsey and Horan, 2018; Butkowski et al., 2019) in addition to traditional media and face-to-face interactions.

In support of objectification theory, both interpersonal (Lindberg et al., 2007; Fairchild and Rudman, 2008; Hill and Fischer, 2008) and mediated (Aubrey, 2006, 2007) exposure to sexual objectification have been shown to increase women's self-objectification (i.e., internalization of the objectifying gaze). Self-objectification is theorized to increase negative affect and to impair cognitive performance, primarily through the consumption of attentional resources (Fredrickson and Roberts, 1997). An increasing body of empirical evidence supports the idea that self-objectification results in decrements in cognitive performance, which we define here as critical reasoning and/or logical reasoning ability, in a variety of contexts. Several possible mechanisms behind the theorized relationship between self-objectification and cognitive performance have been proposed, among them cognitive load associated with appearance monitoring, negative self-conscious emotions (Fredrickson and Roberts, 1997), actual/ideal self-discrepancies (Quinn et al., 2011), gender schema activation, and stereotype threat (Kahalon et al., 2018b). The objective of this review is twofold: (a) to examine the strength of the evidence for a relationship between self-objectification and cognitive functioning, and (b) to evaluate potential mechanisms behind such a relationship. The heterogeneity of the literature surrounding self-objectification and cognitive performance, however, precluded a meta-analytic approach. Therefore, narrative synthesis (Ryan, 2013) was employed to describe and evaluate the results of the studies collected.

## Conceptualizations of Self-Objectification

While self-objectification is often narrowly defined as the adoption of a third-person perspective on the body, the originators of objectification theory define self-objectification as occurring when individuals “treat themselves as objects to be looked at *and* evaluated” (Fredrickson and Roberts, 1997, p. 177; italics added). According to this definition, the adoption of a third-person perspective on the body is a necessary but not sufficient condition for self-objectification. In addition to the perspectival shift from first-person to third-person, self-objectification requires the adoption of an evaluative, appearance-based self-construal.

Self-objectification differs from self-sexualization in that the latter refers to intentional engagement in behaviors designed to enhance one's sexual appeal and/or attract sexual attention (Zurbriggen, 2013). Given that appearance ideals are sexualized, self-objectification constitutes an evaluation of one's own perceived sexual appeal. However, self-objectification does not describe intentional behavior, as self-sexualization does; rather, it constitutes the adoption of an objectified self-perspective. Consequently, while the two constructs are often conflated, it is entirely possible for an individual to self-objectify without self-sexualizing or to self-sexualize without self-objectifying.

In the sexuality studies literature, both self-objectification and self-sexualization have been represented as potentially humanizing and empowering, particularly among those who have not been deemed conventionally attractive. In such contexts, self-objectification is seen as affording a pleasurable experience of sexual desirability that is otherwise inaccessible within the dominant culture. We are mindful of the distinction between self-objectification that is claimed as a radical subversion of appearance ideals and self-objectification in which the body is measured against culturally-dominant appearance ideals. This review focuses on the latter experience of self-objectification and its potential harms.

Objectification theory holds that self-objectification is both a state and a trait (Fredrickson and Roberts, 1997). *State self-objectification* (SSO) refers to the momentary, situationally activated internalization of the objectifying gaze, while *trait self-objectification* (TSO) refers to the frequency with which the state of self-objectification is experienced and its importance to self-construal. However, as Moradi and Huang (2008) have argued, the term “trait” is typically ascribed to biologically-based characteristics that remain stable over time. Self-objectification has been linked to environmental rather than genetic variables (e.g., Hill and Fischer, 2008), and demonstrates significant age-related instability, with young women exhibiting higher self-objectification than older women (Tiggemann and Lynch, 2001). While the term “trait” does not signify total immutability, it typically connotes relative consistency over time, at least as it has become commonplace in the literature on individual differences (c.f. Klimstra et al., 2013). Likewise, the term “trait” does not signify exclusively biological origins, yet it is usually applied to attributes that are perceived as originating primarily from biological, rather than environmental, causes (c.f. Allport's original conception of traits, 1937, see especially, pp. 295 and 559). Therefore, the trait view of self-objectification obfuscates its sociocultural origin and exaggerates its stability. At the same time, substantial individual variability in the extent to which self-objectification is self-defining (Noll and Fredrickson, 1998) validates the conceptualization of self-objectification as an individual difference variable. Thus, we propose the term *chronic self-objectification* (CSO) in lieu of TSO. The language of CSO recognizes individual differences in self-objectification without obscuring their sociocultural origins.

Both CSO and SSO are relevant to cognitive performance, as CSO is hypothesized to increase vulnerability to SSO (Gay and Castano, 2010). Therefore, women high in CSO are likely to suffer greater cognitive decrements in response to objectifying environmental cues. Such cues might include the objectifying gaze, appearance commentary, exposure to objectifying media, or the mere presence of objects that render appearance salient, such as mirrors and scales. In a cultural milieu of sexual objectification and stringent appearance ideals, any reference to physical appearance, however seemingly innocuous, may trigger cognitions associated with self-objectification in women, especially women high in CSO.

## Adaptiveness of Self-Objectification

Women's conformity to appearance ideals is more strongly correlated with social (Feingold, 1990; Crosnoe et al., 2008) and economic (Register and Williams, 1990; Maranto and Stenoien, 2000) outcomes than is men's conformity to appearance ideals. Justifiably, then, women associate conventional attractiveness with social and economic success (Engeln-Maddox, 2006). In effect, conventional attractiveness constitutes a powerful form of “currency” in both social and economic domains (Fredrickson and Roberts, 1997). In light of the evidence that women's conformity to appearance ideals influences others' perceptions and treatment of them, it may be adaptive for women to function as their own first “surveyors” (using Berger's term, 1972). Insofar as appearance monitoring leads women to alter their appearance in accord with cultural ideals, it may improve their socioeconomic outcomes.

At the same time, appearance monitoring is likely to consume cognitive resources, impairing women's ability to perform on cognitive tasks (Fredrickson and Roberts, 1997). Women high in CSO may be unable to “turn off” appearance monitoring in situations in which it is no longer adaptive (e.g., test taking). Moreover, appearance monitoring is unlikely to occur independently of objectifying cognitions in cultures in which sexual objectification is prevalent (Fredrickson and Roberts, 1997). Consequently, *pragmatic* appearance monitoring (c.f. Snyder, 1987), which may also serve other ends, may readily turn into compulsive appearance monitoring to serve exclusively appearance-related goals. Self-objectification may thus serve to hijack women's aspirations. For instance, McKenney and Bigler (2016) found that adolescent girls high on internalized sexualization, a construct similar to CSO, reported lower grades and standardized test scores than did their peers, and prioritized appearance monitoring over studying in a behavioral task.

In sum, women and girls find themselves in a lose-lose situation in which appearance monitoring and lack of appearance monitoring both garner negative consequences. Insofar as the behavioral components of appearance monitoring (e.g., hair and makeup maintenance, selection of flattering clothes) increase perceptions of attractiveness, women's socioeconomic outcomes are likely improved. At the same time, appearance monitoring may impede women's performance through the monopolization of cognitive resources. Moreover, appearance monitoring is likely to activate self-objectification, which may further impede performance and fundamentally undermine women's aspirations.

## The Gender Gap in Cognitive Test Performance

A consistent gender gap favoring boys and men has been documented for the mathematics section of the Scholastic Aptitude Test (SAT), the Advanced Placement (AP) calculus test, the quantitative section of the Graduate Record Exam (GRE) (Niderle and Vesterlund, 2010), and the Graduate Management Admission Test (GMAT) (Educational Testing Service, 2001). While mean score differences are slight, the gender gap becomes pronounced at the right tail of the distribution, with many more boys and men scoring high on measures of quantitative reasoning than girls and women (Niderle and Vesterlund, 2010). As math scores significantly predict future income (Altonjii and Blank, 1999; Murnane et al., 2000), explicating the gender gap in quantitative test performance is critical to women's socioeconomic advancement.

Since self-objectification is theorized to activate gender schemas (Kahalon et al., 2018b), it may enhance awareness of stereotypes surrounding gender and intelligence. Research has demonstrated that reminding women of gender stereotypes surrounding math (Spencer et al., 1999; Quinn and Spencer, 2001) or merely reminding women of their gender (Shih et al., 1999) compromises women's math performance. While gender stereotypes remain particularly powerful in quantitative domains, more general stereotypes surrounding gender and intelligence may compromise women's performance in all domains. This supposition is supported by the finding that male students retain a slight advantage even on the verbal sections of the SAT and GRE (Educational Testing Service, 2001). To the extent that self-objectification increases the salience of gender and thus the accessibility of stereotypes surrounding gender and intelligence, self-objectification may compromise women's cognitive performance in all subject areas, albeit to varying degrees.

Additionally, self-objectification necessitates the adoption of an outside observer's perspective on the body. This “doubling” of attention (Calogero, 2011a) is likely to consume cognitive resources, resulting in performance decrements. Finally, the evaluative dimension of self-objectification necessitates self-comparison with appearance ideals. The narrow and rigid nature of Western appearance ideals is likely to produce actual-ideal self-discrepancies for women in Western cultures when such ideals are made salient. Actual-ideal self-discrepancies are associated with negative self-conscious emotions such as shame (Castonguay et al., 2012) and anxiety (Sabiston et al., 2005), which in turn may impact cognitive performance. Therefore, self-objectification may influence cognitive functioning through multiple possible pathways, cognitive, social, and affective.

## Method

This systematic review of the literature surrounding self-objectification and cognitive performance was conducted in accordance with Cochrane methodologies (Higgins and Green, 2011). An unpublished protocol was formulated prior to beginning the review, the contents of which are presented in the Supplementary Table 1.

### Eligibility Criteria

Studies were deemed eligible for inclusion if they (a) quantitatively investigated the relationship between SSO and cognitive performance using valid and reliable measures, (b) were published in a peer-reviewed journal between 1997 and 2019, inclusive, and (c) were available in English. Studies were required to include an experimental manipulation designed to activate SSO, a manipulation check to verify that SSO was activated, and a cognitive test. Both the manipulation check and cognitive test were required to have demonstrated reliability and validity. Only experimental studies were considered for inclusion in order to evaluate the evidence for a causal relationship between self-objectification and cognitive performance.

The year 1997 was chosen because Fredrickson and Roberts' pioneering article, “Objectification Theory: Toward Understanding Women's Lived Experiences and Mental Health Risks” was published in that year. Prior to “Objectification Theory,” scholars lacked a common vocabulary for the constructs of objectification and self-objectification. In light of the heterogeneity of the literature surrounding self-objectification and cognitive performance, the common definition of self-objectification, introduced in Fredrickson and Roberts (1997), was considered central to the meaningful synthesis of the findings.

### Search Strategy

The following databases were utilized to identify relevant articles: PsycINFO, Medline, Psychology, and Behavioral Sciences Collection, PubMed, Annual Reviews/Social Science Journals, and JSTOR Arts and Sciences IV Collection. The search strategy consisted of the combination of the concept of self-objectification, defined as the adoption of an objectifying self-perspective, with the concept of cognitive performance, defined as critical reasoning ability. Key terms were *objectification, self-objectification, objectification theory, body as object, cognitive performance, cognitive functioning, cognitive load, cognitive resources, attentional resources*, and *cognition*. Keywords were selected based on relevance to critical reasoning ability, as the explication of the gender gap in standardized testing holds particular significance for women's socioeconomic mobility. Consequently, social cognition was excluded from consideration in this review.

### Study Selection

All studies retrieved via keyword search were downloaded into the research organizing platform Zotero. After removal of duplicates, abstracts were reviewed. When abstracts indicated that studies met the eligibility criteria, full texts were reviewed. Full text screening produced the final sample of the included studies.

### Data Collection

Characteristics of included studies were documented in a Microsoft Excel spreadsheet. Information extracted included (1) the country in which the study was conducted, (2) the sample size, (3) the gender composition of the sample, (4) the racial composition of the sample, (5) the mean and standard deviation of participants' ages, (6) the population from which the sample was drawn, (7) the experimental manipulation designed to activate SSO, (8) the manipulation check used to measure SSO, (8) the cognitive test, (9) main findings, and (10) quality rating (see Supplementary Table 1).

### Study Quality

Study quality was evaluated using the AXIS assessment method (Downes et al., 2016), a critical appraisal tool designed to assess the value of cross-sectional studies. The AXIS assessment includes 20 questions to guide critical appraisal of study quality and reporting transparency. Items include, “Were the methods (including statistical methods) sufficiently described to enable them to be repeated?” and, “Were the authors' discussions and conclusions justified by the results?” Three possible answers are provided for each question: *yes, no*, or *don't know*. *Yes* answers are counted as 1, while *no* and *don't know* answers are counted as 0. Studies are thus assigned a quality rating out of 20. The AXIS does not present guidelines for the interpretation of quality scores. Therefore, no specific AXIS rating was specified as an eligibility requirement. However, the shortcomings of lower scoring studies are thoroughly discussed.

## Results

### Study Selection

Keyword searches retrieved 1,779 articles. After abstract screening, 22 were deemed eligible for full text review. Ten studies were eliminated during full text review because they omitted manipulation checks to verify that SSO was activated (Kiefer et al., 2006; Logel et al., 2009, Studies 2, 3, and 4; Gervais et al., 2016; Pacilli et al., 2016, Studies 1 and 2; Baker et al., 2017, Study 3; Kahalon et al., 2018a, Studies 1 and 2). One study was eliminated because it included an indirect manipulation check to verify that SSO was activated (the Appeal of Sex Questionnaire, Gay and Castano, 2010). One study was eliminated because it used a manipulation check that had not been validated (Garcia et al., 2016). Finally, one study was eliminated because it used a cognitive test that had not been validated (Aubrey and Gerding, 2015). Therefore, nine studies remained after full texts were examined in accord with eligibility criteria.

### Study Characteristics

All studies were published in or after 1998 and employed cross-sectional methodologies. Five studies included only female participants (Gapinski et al., 2003; Quinn et al., 2006; Tiggemann and Boundy, 2008; Kozak et al., 2014; Guizzo and Cadinu, 2017). One study included only male participants (Baker et al., 2017, Study 2). Three studies included both male and female participants (Fredrickson et al., 1998, Study 2; Hebl et al., 2004; Gervais et al., 2011). All studies were conducted in Western countries (USA, Canada, Italy, Australia) and all studies drew from college populations. Study characteristics are presented in detail below, along with quality ratings assigned using the AXIS critical appraisal tool. AXIS ratings ranged from 11 to 16 (out of 20) with a modal score of 14 (see Supplementary Table 1, rightmost column).

### Operationalization of Self-Objectification

Seven studies (Fredrickson et al., 1998, Study 2; Gapinski et al., 2003; Hebl et al., 2004; Quinn et al., 2006; Tiggemann and Boundy, 2008; Kozak et al., 2014; Baker et al., 2017, Study 2) administered the modified 20 Statements Test (TST; Fredrickson et al., 1998) as a manipulation check to verify that SSO was activated. The TST has demonstrated high reliability and validity (Fredrickson et al., 1998). Instructions read: “In the 20 blanks below please make up to 20 different statements about yourself to complete the sentence, ‘I am ______.’ Complete the statements as if you were describing yourself to you, not to someone else.” According to the procedure devised and validated by Fredrickson et al. (1998), independent coders categorize statements in one of six groups: (a) body shape and size (e.g., “I am tall”), (b) other physical appearance (e.g., “I am blonde”), (c) physical competence (e.g., “I am in shape”), (d) traits or abilities unrelated to the body (e.g., “I am extraverted”), (e) states or emotions (e.g., “I am tired”), or (f) miscellaneous (e.g., “I am a New Yorker”). The number of statements in categories (a) and (b) constitute participants' SSO scores. Therefore, the TST operationalizes self-objectification as the extent to which the self is defined by the body.

This operationalization omits direct indices of the adoption of the third-person perspective on the body. While appearance valuation and appearance monitoring would appear likely to co-occur in a cultural milieu of objectification and rigid appearance ideals, it is important to note that the TST presupposes this association. By neglecting to explicitly investigate the adoption of the third-person gaze, the TST diverges from the definition of self-objectification presented by the originators of objectification theory (Fredrickson and Roberts, 1997), simplifying the construct.

Additionally, operationalizing self-objectification as the extent to which the self is defined by the body omits the evaluative dimension of self-objectification. Self-objectifying individuals view themselves as visual objects to be evaluated based on their attractiveness (Fredrickson and Roberts, 1997). While the TST counts physical self-descriptions (e.g., “I am fat”) toward SSO, such self-descriptions do not inherently include attractiveness judgments. It is possible for individuals to define themselves largely by their bodies without self-evaluations of attractiveness. While appearance valuation would appear likely to co-occur with evaluative self-directed attention in a cultural milieu of objectification, the TST again presupposes this association.

Omitting direct indices of evaluative appearance monitoring risks overestimating self-objectification. Evolutionary psychologists have generated substantial evidence, mostly among college students, that women's appearance is valued by men to a greater degree than men's appearance is valued by women cross-culturally (Buss, 1989; Conroy-Beam et al., 2015). By contrast, self-reported objectification of women varies by country as a function of westernization (Loughnan et al., 2015). Therefore, objectification and valuation of appearance are not synonymous. Correspondingly, self-objectification and appearance valuation cannot be considered interchangeable constructs.

Two studies (Gervais et al., 2011; Guizzo and Cadinu, 2017) employed the Objectified Body Consciousness Scale-Surveillance (OBCS-S; McKinley and Hyde, 1996) to verify that SSO was activated. The OBCS-S has demonstrated high reliability and validity across gender and ethnic groups (Calogero, 2011b). Unlike the TST, the OBCS-S taps evaluative appearance monitoring with items such as, “I often worry about whether the clothes I am wearing make me look good,” and, “I rarely compare how I look with how other people look” (reverse scored). However, the OBCS-S originated as a measure of CSO. Therefore, the OBCS-S is designed to investigate the extent to which individuals engage in habitual self-objectification, not the extent to which self-objectification is situationally activated. A modified version of the OBCS-S designed to measure SSO has been developed in which participants are instructed to report their experiences “in this precise moment” (Guizzo and Cadinu, 2017). Nevertheless, the content of OBCS-S items arguably remains better suited to the assessment of CSO than SSO.

### Induction of State Self-Objectification

Five methods were utilized to activate SSO. Participants were exposed to an objectifying advertisement (Baker et al., 2017, Study 2), a room with appearance-related objects (i.e., mirrors, scales, and magazine covers; Tiggemann and Boundy, 2008), an objectifying gaze from a trained confederate of the opposite sex (Gervais et al., 2011; Guizzo and Cadinu, 2017), an appearance-related comment (Gapinski et al., 2003; Tiggemann and Boundy, 2008), and/or clothing with a high degree of body exposure (Fredrickson et al., 1998, Study 2; Gapinski et al., 2003; Hebl et al., 2004; Quinn et al., 2006; Kozak et al., 2014). In order to evaluate the strength of the relationship between SSO and cognitive performance, it is necessary to evaluate the efficacy of each SSO induction.

In line with objectification theory, SSO was successfully activated by requesting participants to try on a swimsuit (vs. sweater) (*p* < 0.01 for all four studies; Fredrickson et al., 1998, Study 2; Gapinski et al., 2003; Hebl et al., 2004; Quinn et al., 2006) and by exposing participants to objectifying media (*p* = 0.01, *d* =.41; Baker et al., 2017, Study 2). However, no other methods were efficacious in inducing SSO. Many potential confounds may account for the null results obtained for the effects of alternative SSO inductions (e.g., exposure to the objectifying gaze, appearance-related objects, and appearance-related comments). These must be addressed study by study.

Gervais et al. (2011) trained confederates to gaze at participants in a sexualized manner (i.e., scanning their bodies and glancing repeatedly at their chests) and to provide objectifying written feedback [i.e., “(participant's name) was looking good”]. A manipulation check using a modified version of the Interpersonal Sexual Objectification Scale (ISOS; Kozee et al., 2007) determined that perceptions of objectification were indeed higher in the objectifying condition (*p* < 0.004, η^2^ = 0.04). However, no differences in SSO were observed between conditions. This null finding is likely attributable to the use of the original version of the OBCS-S, a measure designed to assess CSO rather than SSO. A main effect of gender rather than condition was observed for OBCS-S scores, with women scoring higher than men (*p* < 0.001, η^2^ = 0.10), a finding in line with research that women exhibit higher rates of CSO than do men (Moradi and Huang, 2008). Hence, the null relationship between condition and SSO here is likely attributable to measurement error or chance.

Additionally, the manipulation employed by Gervais et al. (2011) appears ill-suited to activate SSO in men, as the chest area is more sexualized for women than for men. ISOS scores did not vary based on gender, ostensibly legitimizing the manipulation as equally objectifying for both genders. However, one of the items in the ISOS asks about how often participants noticed their interaction partner staring at their chest, and another item asks about how often participants noticed their interaction partner staring at their body generally. Given that women's chests, specifically, and their bodies, generally, are sexualized to a greater degree than are men's, such items risk establishing false equivalencies between the objectification experienced by men and women in response to the same behaviors. Therefore, the inclusion of the ISOS may have perpetuated the gender inequality of the SSO manipulation while appearing to validate the manipulation.

Guizzo and Cadinu (2017) manipulated SSO by employing either a same-sex or opposite-sex experimenter to photograph participants from the neck down. No differences in SSO were observed between conditions even though SSO was measured using the appropriately modified version of the OBCS-S. It is possible that photographing participants from the neck down produced a ceiling effect of SSO; that is, it activated such a high degree of SSO that the gender of the photographer failed to increase SSO further. It is also possible that low degrees of body exposure reduced the salience of the photographer's gender, as the study was conducted during cold weather. In a pilot test conducted during warm weather, a main effect of photographer gender on SSO was observed (*p* = 0.041, η^2^ = 0.03). Therefore, greater body exposure may have been necessary to elicit an effect of photographer gender on SSO.

When SSO was manipulated with an appearance comment, no effect of condition on SSO was observed (Tiggemann and Boundy, 2008). However, the comment (i.e., “I like your top, it looks good on you”) targeted women's clothing rather than their bodies and it came exclusively from female experimenters. Therefore, both the content of the comment and the gender of the speaker constitute potential confounds. Objectification requires a reduction of person to body (Fredrickson and Roberts, 1997), while the comment employed by Tiggemann and Boundy (2008) merely conveys a favorable impression of one's clothing.

When SSO was manipulated using environmental cues (i.e., mirrors, scales, and magazine covers), only women high in CSO exhibited a difference in SSO between conditions (Tiggemann and Boundy, 2008). Therefore, environmental cues appear insufficient to reliably activate SSO. However, Tiggemann and Boundy's (2008) findings underscore the heightened vulnerability of high CSO women to SSO activation. For women who habitually view themselves through an objectifying lens, mere exposure to appearance-related objects may induce SSO.

Finally, SSO was manipulated by randomly assigning women to try on either a fitted tank top or a sweatshirt (Kozak et al., 2014). Oddly, the authors do not report if the main effect of clothing condition on SSO was calculated, suggesting that the relationship was not significant. However, a significant interaction of shirt and posture was reported such that women who sat upright in the tank top condition scored higher on SSO than women who sat in a slouched position (*p* = 0.009, *d* = 0.88), while no such interaction was observed in the sweatshirt condition. The authors attribute this finding to the emphasis on the chest area placed by sitting upright in a shirt with a high degree of body exposure.

In sum, only the swimsuit/sweater and objectifying media manipulations successfully activated SSO. However, significant confounds undermine the null findings of the objectifying gaze and appearance comment manipulations in the other studies. Research has identified a relationship between CSO and the frequency of women's self-reported experience of the objectifying gaze (Hill and Fischer, 2008). Given the relationship between SSO and CSO, it is therefore likely that objectifying gaze is indeed sufficient to activate SSO. Likewise, evidence indicates that self-reported frequency of receiving evaluative body commentary from men predicts CSO in women (Fairchild and Rudman, 2008; Hill and Fischer, 2008). Thus, appearance comments may successfully activate SSO if they are (a) evaluative, (b) body-focused rather than clothing-focused, and (c) spoken by a man to a woman. However, ethical concerns prohibit exploration of the effects of such appearance comments on SSO. Moreover, both the objectifying gaze and appearance comment manipulations are associated with a greater number of potential confounds than the swimsuit/sweater and objectifying media manipulations (e.g., almost inevitable alterations in confederates' body language and tone of voice).

The swimsuit/sweater and objectifying media manipulations did not differ in terms of statistical significance. In terms of external validity, however, the objectifying media manipulation appears to surpass the swimsuit/sweater manipulation, as individuals are likely to spend more time exposed to objectifying media than wearing swimsuits or clothing with equivalent body exposure. In light of the increased media objectification of men (Rohlinger, 2002), the objectifying media manipulation promises high external validity for both genders. Additionally, the objectifying media manipulation appears to elicit self-comparison to appearance ideals more directly than the swimsuit/sweater manipulation does. Therefore, the objectifying media manipulation is recommended for future research into the effects of self-objectification.

### Gender and Self-Objectification

Objectification theory posits that women are more vulnerable to SSO, as women are exposed to more frequent instances of both interpersonal and media objectification (Fredrickson and Roberts, 1997). However, two out of three studies including both male and female participants found no gender differences in SSO activation (Fredrickson et al., 1998; Hebl et al., 2004). The one study that did observe differences (Gervais et al., 2011), was undermined by a significant confound: the manipulation of the objectifying gaze focused on participants' chests, resulting in unequal objectification of men and women. Furthermore, exposure to an objectifying ad activated SSO in an all-male sample (Baker et al., 2017, Study 2). Thus, men appear to be vulnerable to SSO to a greater degree than theorists have supposed. However, it is important to remember that women and girls remain the principal targets of objectification, both in the media (Aubrey and Frisby, 2011; Conley and Ramsey, 2011) and in person (Garcia et al., 2016; Gervais et al., 2018). Hence, frequent experiences of SSO may result in poorer performance outcomes for women.

The evidence for decrements in men's cognitive functioning in conjunction with SSO is somewhat mixed. Fredrickson et al. (1998) found no differences in men's math performance between conditions (swimsuit vs. sweater), despite elevated SSO. By contrast, women marginally underperformed in the swimsuit condition (*p* = 0.056). Protective factors, such as positive stereotypes of men's mathematical ability, may have mitigated the effects of SSO on men's performance. Conversely, Hebl et al. (2004) reported decrements in both men's and women's math performance in the swimsuit condition (*p* < 0.01, *r*^2^ = 0.22). No interaction was observed between gender and condition, although women scored lower than men overall (*p* < 0.01).

This discrepancy may be attributable to the SSO induction; Fredrickson et al. (1998) provided men with swim trunks while Hebl et al. (2004) provided men with Speedos. The body exposure produced by Speedos renders them a more suitable equivalent to the tight-fitting swimsuits provided to women in both studies. However, outside the lab, men are more likely to wear swim trunks than Speedos. Therefore, researchers were forced to either sacrifice external validity for an equivalent SSO induction, or to sacrifice an equivalent SSO induction for external validity.

Instead, Baker et al. (2017, Study 2) utilized an SSO induction with high external and internal validity: men either viewed an advertisement that objectified a male model demonstrating the muscular ideal or a neutral, non-objectifying advertisement. Men exposed to the objectifying advertisement underperformed on a visual-spatial task (*p* = 0.019, *d* = 0.41). Therefore, Baker et al. (2017, Study 2) present compelling evidence that men's cognitive functioning is compromised by SSO. At the same time, greater media objectification and interpersonal objectification of women indicate that women experience SSO more frequently, potentially culminating in poorer outcomes.

Indeed, women's cognitive performance was compromised in every study that successfully activated SSO (Fredrickson et al., 1998; Hebl et al., 2004; Quinn et al., 2006) except for one (Gapinski et al., 2003). The one study that failed to observe decrements in women's performance despite increased SSO contained a significant confound, however. Half of the participants overheard a self-deprecating appearance-related remark from a female confederate (i.e., “Do you have to try this thing on, too? I look totally fat in this!”). Gapinski et al. (2003) neglected to calculate a main effect of comment condition (appearance comment vs. neutral comment) on SSO or the effect size of the difference in SSO between swimsuit and sweater conditions. In the swimsuit condition, overhearing the self-deprecating remark may have meaningfully shifted participants' focus away from their own bodies, resulting in decreased SSO. Consequently, the comment manipulation may have decreased the difference in SSO between clothing conditions.

Moreover, it is possible that overhearing the self-deprecating comment increased positive affect and state self-esteem, which in turn buffered the effects of SSO on performance for women in the swimsuit condition. Participants did not view the confederate who made the remark, as they were trying on swimsuits in separate cubicles. Therefore, regardless of appearance, all participants may have made downward social comparisons with the female confederate. Downward social comparisons are associated with increased positive affect (Bogart et al., 2004), which is positively related to cognitive performance (Isen, 2001; Yang et al., 2013). Hence, the comment manipulation thoroughly undermines the import of Gapinski et al.'s (2003) null finding.

Gervais et al.'s (2011) findings add to the evidence that SSO compromises women's cognitive performance. While SSO was assessed using a measure of CSO, and therefore no differences in SSO were observed between conditions, the ISOS manipulation check confirmed that women experienced more objectification in the objectifying gaze condition. Women in the objectifying gaze condition performed worse on a math test than women in the control condition (*p* < 0.011, η^2^ = 0.04). The effect size was fairly small, as the sample included only 67 women. Nevertheless, even in an extremely low-powered sample, decrements in women's cognitive functioning were observed in conjunction with the experience of objectification. In sum, the small body of literature surrounding SSO and cognitive performance suggests that SSO produces deleterious effects on women's cognitive functioning, with significant potential repercussions for women's socioeconomic standing.

### Race/Ethnicity and Self-Objectification

Only one study (Hebl et al., 2004) investigated the relationship between race/ethnicity and self-objectification. A significant interaction between race/ethnicity and CSO was observed, with Hispanic participants scoring highest and African American participants scoring lowest. However, there were no differences in SSO across race/ethnicity. Additionally, there was no interaction between condition and race/ethnicity on math performance, indicating that all ethnic groups experienced equivalent decrements in the swimsuit condition. On the other hand, the condition × gender × ethnicity/race interaction was not calculated, and the reported performance decrements in the swimsuit condition (7% for Caucasian men compared with 31% and 20% for Hispanic and African American men, respectively) suggest that White men were shielded somewhat from the adverse effects of SSO on cognitive functioning. The diminished influence of SSO on White men indicates that positive stereotypes surrounding intellectual ability may buffer the effects of SSO.

On the whole, the empirical neglect of the relationship between race/ethnicity and SSO is striking. SSO, racial stereotypes, and gender stereotypes may interact to produce unique effects for each identity group. For women of color, SSO may activate both gender schemas and racial schemas, increasing the salience of both gender stereotypes and racial stereotypes. The deleterious effects of SSO on cognitive functioning may thus be compounded by stereotype priming or stereotype threat. Consequently, women of color may be at particularly high risk for performance decrements in conjunction with SSO.

### Stereotype Priming and Stereotype Threat

Stereotypes of intellectual inferiority may be either internalized (resulting in decreased effort on cognitive tasks) or externalized (resulting in increased effort as well as increased cognitive load) (Jaimeson and Harkins, 2011). Stereotype priming appears to produce stereotype-consistent behavior, while stereotype threat appears to motivate attempts to disconfirm stereotypes (Jaimeson and Harkins, 2011). However, both stereotype priming and stereotype threat are associated with performance decrements (Jaimeson and Harkins, 2011). To the extent that SSO increases the salience of group membership, it would appear to increase the salience of stereotypes.

Both stereotype priming and stereotype threat are associated with the activation of schemas surrounding group membership. This is compelling evidence that SSO is linked to gender schema activation. For example, Saguy et al. (2010) found that women videotaped from the neck down talked less than women videotaped from the neck up, suggesting increased conformity to traditional gender roles privileging appearance over speech. Additionally, both SSO and CSO have been linked to gender-specific system justification and decreased willingness to engage in gender-based social activism (Calogero, 2017). Such findings suggest that SSO triggers gender schema activation, which may in turn increase the salience of stereotypes. Schema retrieval alone is theorized to increase cognitive load (Aubrey and Gerding, 2015). As gender schema activation and stereotype activation are inextricably linked (Shih et al., 1999), the latter may compound the effects of the former.

The association between SSO and gender role conformity suggests that stereotype priming is more strongly linked to SSO than it is to stereotype threat. An SSO induction such as trying on a swimsuit may prime stereotypes by simply reminding participants of their gender, resulting in stereotype-consistent behavior (i.e., decreased effort). At the same time, the context of cognitive testing may serve to bring negative stereotypes to the forefront of participants' awareness, resulting in attempts to disprove stereotypes (i.e., by increased effort). Therefore, it is unclear whether stereotype priming or stereotype threat is more strongly implicated in the relationship between SSO and cognitive performance. No study addressed stereotype priming as a possible mechanism, but several studies addressed (and claimed to find evidence against) stereotype threat.

Fredrickson et al. (1998, Study 2) argue that the discrepancy between women's math scores in the swimsuit and sweater conditions rules out stereotype threat, as it is, “improbable that women in the sweater condition were unaware of the gender stereotype regarding math” (p. 280). However, theorists of stereotype threat have posited that the phenomenon is best understood as a spectrum rather than a binary (Schmader et al., 2008). While both women in swimsuits and women in sweaters performed in stereotype-relevant contexts, women in swimsuits may have experienced stereotype threat *to a greater degree*. Increasing the salience of gender has been shown to increase stereotype threat (Shih et al., 1999; Spencer et al., 1999; Quinn and Spencer, 2001). As wearing a swimsuit exposes the body, it may thus increase the salience of gender. Therefore, women in the swimsuit may have experienced both greater SSO and greater stereotype threat than women in the sweater condition.

Quinn et al.'s (2006) failure to find support for stereotype threat likewise relies upon an “all or nothing” conceptualization of it. Quinn et al. (2006) attempted to rule out stereotype threat through the use of an ostensibly gender-neutral Stroop task in lieu of a math test. Finding that women in swimsuits exhibited slower reaction times than women in sweaters, the authors concluded that the deleterious effect of SSO on cognitive functioning occurred independently of stereotype threat. However, gender stereotypes concern intelligence generally, not merely mathematical ability (Petrides et al., 2004; Verniers and Martinot, 2015). Consequently, *any* cognitive task may elicit stereotype threat in women. Of course, not all cognitive tasks are likely to elicit stereotype threat to the same degree. It is probable that women experience greater stereotype threat during math tests than during Stroop tests due to particularly entrenched stereotypes surrounding women's ability in quantitative domains. Nevertheless, to dismiss stereotype threat altogether as an influence on women's Stroop performance is premature.

Hebl et al. (2004) present a much more compelling challenge to the involvement of stereotype threat in women's underperformance in the swimsuit condition, as no interaction between gender and condition was observed in their study. At the same time, there was a significant effect of gender on performance, such that women scored lower than men overall. Therefore, Hebl et al.'s (2004) findings suggest that stereotype threat occurred independently of SSO. Their data appear to contradict the evidence that SSO activates gender schemas, which enhance awareness of gender stereotypes. Further research is clearly necessary to unravel the relationship between SSO and stereotype activation, and to determine whether stereotype priming or stereotype threat is more strongly implicated in cognitive decrements.

### Negative Self-Conscious Emotions

Negative self-conscious emotions such as shame and anxiety constitute additional potential mechanisms behind the relationship between SSO and cognitive performance (Fredrickson and Roberts, 1997). Negative self-conscious emotions are theorized to arise from actual-ideal self-discrepancies in conjunction with SSO (Fredrickson and Roberts, 1997). The adoption of an observer's perspective on the body would appear to increase awareness of cultural appearance ideals, while the identity reduction from person to body would appear to increase the perceived importance of appearance ideals. Given the narrow and rigid nature of such ideals in Western countries (c.f. Berger, 1972), heightened awareness and valuation of appearance ideals is likely to result in actual-ideal self-discrepancies. Emotions such as shame and anxiety may arise from self-discrepancies, compromising cognitive functioning.

Fredrickson et al. (1998) found that trying on a swimsuit elevated body shame in women (*p* < 0.05) but not in men. Gender differences in negative self-conscious emotions in conjunction with SSO are not surprising given that appearance ideals are generally more stringent for women than for men, and conformity to appearance ideals is a stronger determinant of social and economic success for women than it is for men (Fredrickson and Roberts, 1997). Therefore, women likely experience greater actual-ideal appearance discrepancies and accord greater weight to actual-ideal appearance discrepancies than do men, resulting in greater negative affect in conjunction with SSO. However, Fredrickson et al. (1998) found that the swimsuit manipulation was associated with increases in general shame (*p* < 0.001) and guilt (*p* < 0.05) for both men and women. Therefore, men do appear to experience negative affect as a result of SSO, but the negative emotions they experience are more generalized rather than body-specific. Additionally, Fredrickson et al. (1998) found that men in the swimsuit condition reported feeling more “sheepish, bashful, shy” than did women (*p* = 0.05), while women reported feeling more “disgust, distaste, revulsion” than did men (*p* < 0.05). Hence, the import of men's negative affect, in conjunction with SSO, appears to be lighter than the import of women's negative affect under the same conditions.

Quinn et al. (2006) replicated Fredrickson et al.'s (1998) finding that the swimsuit manipulation increased body shame in women (*p* < 0.01). Additionally, Gapinski et al. (2003) observed that the swimsuit manipulation increased women's feelings of fear (*p* < 0.05) and humiliation (*p* < 0.05). However, no study has investigated negative self-conscious emotions as mediators or moderators of the relationship between SSO and cognitive performance. Therefore, it remains unclear whether negative self-conscious emotions are implicated in the cognitive decrements observed.

In a re-analysis of Quinn et al.'s (2006) data, Quinn et al. (2011) found no correlation between body shame and Stroop performance. Quinn et al. (2011) conclude that negative self-conscious emotions and cognitive functioning, “do not form a direct causal chain” (p. 14). Instead, the authors posit that actual-ideal self-discrepancies trap women in a “self-regulatory feedback loop.” According to self-regulation theory, self-focus continues when behavioral changes cannot be made to reduce discrepancies, diverting attention away from cognitive tasks. However, the influence of negative self-consciousness emotions cannot be ruled out on the basis of one study. Moreover, the study in question evaluated only the role of body shame. Fear and anxiety may be implicated more strongly than shame in the relationship between SSO and cognitive performance in this context. Therefore, further research is called for to evaluate the role of negative self-conscious emotions in the cognitive decrements associated with SSO.

### Chronic Self-Objectification as a Moderator

Individuals with high CSO appear to experience SSO more frequently and to experience greater decrements in cognitive functioning in conjunction with SSO. Tiggemann and Boundy (2008) found that an environment with appearance-related objects (i.e., mirrors, scales, and magazine covers) only activated SSO in high CSO women. Additionally, high CSO women experienced more body shame in the appearance compliment condition (i.e., “I like your top, it looks good on you”) than low CSO women. Therefore, seemingly innocuous appearance cues suffice to produce negative psychological outcomes for high CSO women.

While Gapinski et al. (2003) neglected to calculate an effect of appearance comment condition on SSO, high CSO women who overheard the comment (i.e., “Do you have to try this thing on, too? I look totally fat in this!”) performed worse on three cognitive tests designed to assess processing speed, logical reasoning, and spatial orientation (*p* < 0.05 on all three). Overhearing such a self-deprecating appearance-related comment may have served to decrease SSO in the majority of women by shifting their focus away from their own bodies, as was argued above. For women high in CSO, however, any appearance comment (self-deprecating or not) may contain the potential to trigger or heighten SSO, as appearance schemas are activated so easily and bound up so closely with objectifying cognitions.

Correspondingly, Guizzo and Cadinu (2017) found that internalization of beauty ideals— an essential component of CSO (Fredrickson and Roberts, 1997)—was linked to decreased performance on a Sustained Attention to Response Task (SART) among women photographed by male experimenters. The SART assesses attentional resources (Robertson et al., 1997). Therefore, internalization of appearance ideals, in conjunction with the male gaze, appears to divert women's attention toward their physical selves and away from the task at hand. Considering that internalization of beauty ideals is a dimension of CSO, Guizzo and Cadinu's (2017) findings appear to support the hypothesized function of CSO as a moderator of the relationship between experiences of objectification and cognitive decrements. As women exhibit higher rates of CSO than men (Fredrickson et al., 1998), the probable moderating role of CSO underscores the importance of gender differences in the relationship between SSO and cognitive performance.

## Discussion

Although small, the body of literature surrounding SSO and cognitive performance presents compelling evidence that SSO compromises cognitive functioning. Declines in women's performance were documented across measures of quantitative reasoning (Fredrickson et al., 1998, Study 2; Hebl et al., 2004) spatial perception (Baker et al., 2017, Study 2), and selective attention (Quinn et al., 2006). The swimsuit manipulation and objectifying media manipulation consistently activated SSO in both women and men, indicating that men possess greater vulnerability to SSO than originally theorized. At the same time, the operationalization of SSO employed by most researchers omitted direct indices of evaluative self-directed attention. Therefore, it is unclear whether SSO manipulations increase self-objectification or merely increase appearance valuation. Greater interpersonal and media objectification of women indicates a closer association between appearance valuation and objectifying self-perspectives in women than in men. However, measures of SSO were associated with cognitive decrements for men as well as for women (Hebl et al., 2004; Baker et al., 2017, Study 2). Therefore, the adoption of an appearance-based self-construal alone may suffice to compromise cognitive functioning.

Appearance monitoring, in particular, has long been implicated in cognitive decrements. However, the two studies that utilized measures of appearance monitoring failed to find differences between conditions. Therefore, it is impossible to evaluate the role of appearance monitoring in the relationship between SSO and cognitive performance. Additional mechanisms behind the relationship between SSO and cognitive performance were largely unexplored. A number of studies linked SSO to negative self-conscious emotions such as shame and guilt (Fredrickson et al., 1998; Gapinski et al., 2003; Quinn et al., 2006). However, no study investigated negative self-conscious emotions as mediators or moderators of the relationship between SSO and cognitive performance. The role of stereotype activation was likewise neglected. Fredrickson et al. (1989) and Quinn et al. (2006) advance the claim that their findings rule out stereotype threat, yet their conceptualization of stereotype threat as “all or nothing” fails to accord with the evidence that stereotype threat may be evoked to varying degrees.

As SSO draws awareness to the body, it also draws awareness to gender and race/ethnicity. Awareness of group membership may trigger stereotype activation (Shih et al., 1999; Spencer et al., 1999; Quinn and Spencer, 2001). Therefore, conceptualizing SSO and stereotype activation as operating independently of one another may mask the connection between SSO, gender, and ethnicity. While no study known to us has investigated the relationship between SSO and racial/ethnic schema activation, substantial evidence supports the association between SSO and gender schema activation (e.g., Calogero, 2017).

Gender schema activation is inextricably linked to gender stereotype activation. Therefore, women induced to self-objectify are likely to experience stereotype priming or stereotype threat, especially in stereotype-relevant contexts such as cognitive testing. Whether stereotype priming or stereotype threat is more strongly implicated in the relationship between SSO and cognitive decrements is unclear. SSO may facilitate stereotype priming, as it reminds individuals of their group membership(s). However, contexts such as cognitive testing may serve to bring stereotypes to the forefront of consciousness, resulting in stereotype threat. Further research is necessary to determine whether SSO results in stereotype-consistent behaviors in cognitive testing contexts (e.g., fewer questions attempted, indicating stereotype priming) or stereotype-inconsistent behaviors (e.g., more questions attempted, indicating stereotype threat).

Stereotypes may constitute both risk factors and protective factors for SSO depending on stereotype content. Positive intellectual stereotypes may serve to mitigate the effects of SSO on cognitive performance. While Hebl et al. (2004) neglected to calculate the condition × gender × ethnicity/race interaction, Caucasian men evidenced smaller cognitive decrements in the swimsuit condition than Hispanic or African American men. Therefore, positive stereotypes associated with dominant group membership may constitute protective factors, buffering the effects of SSO on performance, while negative stereotypes associated with marginalized group membership may constitute risk factors, exacerbating the effects of SSO on performance.

The literature points to CSO as a second important risk factor. Individuals who habitually view themselves through an objectifying lens are more susceptible to SSO activation (Tiggemann and Boundy, 2008) and experience more negative outcomes in conjunction with SSO (Gapinski et al., 2003; Tiggemann and Boundy, 2008). Women exhibit higher rates of CSO than do men (Moradi and Huang, 2008). Therefore, while men are not invulnerable to the deleterious effects of SSO, women are likely to experience them more frequently and more intensely. Consequently, the repercussions of the relationship between SSO and cognitive performance may be greater for women than they are for men. As SSO appears to increase the salience of group membership, women of color may be at highest risk of experiencing cognitive decrements in conjunction with SSO. However, Caucasian women are overrepresented in the literature. Future research should rectify this bias and center those likely to suffer the greatest effects of SSO on cognitive performance.

### Limitations of the Literature

Only two studies (Gervais et al., 2011; Kozak et al., 2014) reported calculations of statistical power, and one of the two (Kozak et al., 2014) reported extremely low power (0.12 to 0.17). Therefore, by Kozak et al.'s (2014) own calculations, their sample size was insufficient to detect anything but a large effect. Thus, only one out of nine studies justified their sample sizes. The failure of the majority of researchers to calculate statistical power undermines the small body of literature surrounding SSO and cognitive performance. Additionally, just over half of the studies included in this review (Hebl et al., 2004; Gervais et al., 2011; Baker et al., 2017, Study 2; Guizzo and Cadinu, 2017; Kozak et al., 2014) reported effect sizes. *P*-values alone offer no insight into the magnitude of observed effects. Therefore, the omission of effect sizes further weakens the reliability of the literature.

In some cases, design flaws compounded shortcomings in statistical reporting. SSO was successfully activated in only a slight majority of studies. In one study (Gervais et al., 2011), the failure of the SSO manipulation may be attributable to measurement error, as the authors administered the original version of the OBCS, which is typically used to assess CSO rather than SSO. The objectifying condition did indeed result in increased perceptions of interpersonal objectification, indicating that SSO was activated. Nevertheless, only five studies documented differences in SSO between conditions, resulting in an extremely small body of literature from which to draw conclusions about the strength of the relationship between SSO and cognitive performance.

Finally, the generalizability of the findings presented in this review may be compromised by the exclusive use of college student samples. As young adults score highest on self-objectification (Tiggemann and Lynch, 2001), it is appropriate to focus on young people in investigations of SSO. However, drawing solely from college populations risks overrepresenting certain racial and socioeconomic groups and underrepresenting others. This potential bias is particularly concerning in light of the possibility that identity groups of various kinds experience both different forms of objectification and different rates of objectification. Correspondingly, different identity groups may vary in both levels of SSO activation and in effects of SSO activation. Therefore, future research should utilize sampling methods that maximize racial and socioeconomic diversity.

### Limitations of the Systematic Review

A number of limitations of this review must be acknowledged. First, the databases utilized only provided access to published studies. Recent reviews indicate strong evidence for publication bias in the field of psychology as a whole (i.e., the disproportionate representation of statistically significant findings in the published literature; Joober et al., 2012). Consequently, studies that observed significant relationships between SSO and cognitive performance may be overrepresented in this review. A second limitation is the lack of a common operationalization of SSO across studies. While the majority of studies operationalized SSO as the number of appearance-related characteristics associated with aspects of identity, two studies operationalized SSO as body surveillance. Such differential conceptualizations of self-objectification impeded the meaningful synthesis of results. Finally, the strict eligibility criteria of this review eliminated a number of studies due to the use of untrialled measures. However, the measures administered in several excluded studies possess high face validity. Therefore, while publication bias would suggest that this review overestimates the strength of the relationship between SSO and cognitive performance, relevant excluded studies suggest that this review might very well underestimate the strength of the relationship.

## Conclusion

The small body of literature surrounding SSO and cognitive performance indicates that SSO is associated with cognitive decrements in both women and men. CSO emerged as a probable moderator of the effect of SSO on cognitive performance. Higher rates of CSO in women, in conjunction with higher rates of interpersonal and media objectification, suggest that the deleterious effects of SSO exert greater influence over women's life outcomes. Studies have largely neglected to investigate potential mechanisms behind the relationship between SSO and cognitive performance. Future research should systematically evaluate the roles of appearance monitoring, actual-ideal self-discrepancies, negative self-conscious emotions, gender schema activation, and stereotype activation. A more precise elucidation of the relationship between SSO and cognitive performance is critical to understanding barriers to women's socioeconomic advancement.

## Author Contributions

LW carried out the analysis and wrote the majority of the article. Both authors listed, however, have made a substantial, direct and intellectual contribution to the work, and approved it for publication.

### Conflict of Interest

The authors declare that the research was conducted in the absence of any commercial or financial relationships that could be construed as a potential conflict of interest.
