# Erratum: Self-Objectification and Cognitive Performance: A Systematic Review of the Literature

**DOI:** 10.3389/fpsyg.2020.00477

**Published:** 2020-03-13

**Authors:** Frontiers Production Office

**Affiliations:** Frontiers Production Office, Frontiers Media SA, Lausanne, Switzerland

**Keywords:** self-objectification, objectification theory, objectified body consciousness, body as object, cognitive performance, critical reasoning ability, cognitive functioning, cognitive load

Due to a typesetting error brackets were removed for point (3) in the introductory text, thus changing the meaning of the sentence. A correction has been made to the 2nd paragraph of the article.

“Objectification theory posits that repeated experiences of sexual objectification socialize girls and women to adopt an evaluative third-person perspective on their bodies (McKinley and Hyde, 1996; Fredrickson and Roberts, 1997). Fredrickson and Roberts (1997) define sexual objectification as, “occur[ing] whenever a woman's body, body parts, or sexual functions are separated out from her person, reduced to the status of mere instruments, or regarded as if they were capable of representing her” (p. 175). Theorists suggest that girls and women are exposed to sexual objectification in three primary ways: (1) direct interpersonal experiences of objectification (e.g., unsolicited appearance commentary), (2) vicarious experiences of other women's objectification (e.g., overhearing men's appearance commentary about other women), and (3) objectified media representations of women (e.g., images in which women's bodies are fragmented) (Fredrickson and Roberts, 1997). All three forms of objectification are now commonly experienced by women on social networking sites such as Instagram, Facebook, and Twitter that have been developed since Fredrickson and Roberts' early work (see, for example, Bell et al., 2018; Ramsey and Horan, 2018; Butkowski et al., 2019) in addition to traditional media and face-to-face interactions.”

The publisher apologizes for this error. The original article has been updated.

