# Supplementary material for: Self-Objectification and Cognitive Performance: A Systematic Review of the Literature
Source: Front Psychol. 2020 Jan 28;11:20. doi: 10.3389/fpsyg.2020.00020 (PMC6997128; doi:10.3389/fpsyg.2020.00020)
Supplement: Supplementary file 1 [file Table_1.DOCX]

Table

*Study characteristics*

| **Study** | **Country** | **Sample size** | **Gender composition** | **Racial composition** | **Age** | **Population** | **SSO**  **manipulation** | **SSO measure** | **Cognitive test** | **Results** | **Quality rating**  **(/20)** |
| --- | --- | --- | --- | --- | --- | --- | --- | --- | --- | --- | --- |
| Baker, Elnakouri, and Blanchard (2017, Study 2) | Canada | 148 | Male only | 54.7% Caucasian,  14.9% Middle  Eastern, 10.1% African Canadian, 8.8% Native  Canadian, 4.7%  indigenous, 6.8% other | *M* = 19.59,  *SD* = 2.09 | College students | Exposure to an objectifying advertisement vs. exposure to a non- objectifying advertisement | TST | Soma puzzle task | Participants in the objectifying advertisement condition scored higher on SSO (*p* = .01, *d* = .41). Participants performed worse on a visual-spatial task in the objectifying advertisement condition *(p* = .019, *d* = .41). | 15 |
| Fredrickson, Roberts, Noll, Quinn, and Twenge (1998,  Study 2) | USA | 82 | 51.2% female,  48.8% male | 83% Caucasian,  6% African  American, 5% Asian American, 2% Hispanic, 4% other | Unspecified | College students | Trying on a swimsuit vs. trying on a sweater | TST | GMAT | Participants in the swimsuit condition scored higher on SSO than participants in the sweater condition (*p* < .01). Women in the swimsuit condition scored higher on body shame (*p* < .05), while men did not. However, both women and men in the swimsuit condition reported feeling more general shame (*p* < .001) and guilt (*p* < .05). Women in the swimsuit condition performed marginally worse on a math test than women in the sweater condition (*p* = .056) while men's performance did not vary between conditions. | 11 |
| Gapinski, Brownell, and LaFrance (2003) | USA | 82 | Female only | 61% Caucasian,  14% Asian  American, 10% African American, 6% Hispanic, 9% other | *M* = 18.69,  *SD* = .96 | College students | Trying on a swimsuit vs. trying on a sweater; overhearing a self- deprecating appearance comment ("fat talk") vs. a non- appearance-related comment ("control talk") | TST | Gestalt Completion Test, Nonsense Syllogisms Test, Surface Development Test, GMAT | Participants in the swimsuit condition scored higher on SSO than participants in the sweater condition (*p* < .01). Participants in the swimsuit condition reported feeling more fearful (*p* < .05) and humiliated (*p* < .05) than participants in the sweater condition. Performance did not vary for any cognitive task based on clothing condition (swimsuit vs. sweater) or talk condition ("fat talk" vs. "control talk"). However, in the "fat talk" condition, participants with high CSO performed worse than participants with low CSO on the Gestalt Completion Test (p < .05), the Nonsense Syllogisms Test (p < .05), and the Surface Development Test (p <  .05). | 13 |
| Gervais, Vescio, and Allen (2011) | USA | 150 | 55.3% male,  44.7% female | 89% Caucasian,  2.7% African  American, .67%  Hispanic, 3.3% Asian American, 1.3% multiracial,  2.7% other | *M* = 19.16,  *SD* = 1.49 | College students | Receiving an objectifying gaze and objectifying written feedback from a trained confederate of the opposite sex vs. receiving a non- objectifying gaze and non- objectifying feedback | OBCS-S | GRE-Q | Participants did not vary in SSO or body shame between conditions. However, participants in the objectifying gaze condition reported increased perceptions of objectification (*p* <  .001, *n ^2^* = .04). There was no effect of condition on body shame, but there was an effect of gender such that women scored higher on body shame than men (*p* < .001, *n ^2^* = .13). Women in the  objectifying gaze condition performed worse than women in the  control condition (*p* < .011, *n ^2^* = .04), while men's performance did not vary between conditions. | 14 |

| Guizzo and Cadinu (2017) | Italy | 107 | Female only | 100% Caucasian | *M* = 21.23,  *SD* = 2.35 | College students and community | Being photographed from the neck down by an opposite-sex vs. same-sex experimenter | OBCS-S | SART | There was no main effect of condition (same-sex experimenter vs. opposite-sex experimenter) on SSO or SART performance. However, for participants in the male gaze condition, internalization of beauty ideals was inversely associated with the Challenge-Skill Balance subscale of the Flow Experience State  Scale (*p* = .001, *r* ^2^ = .19), which was in turn inversely associated  with SART score (*p* < .001, *r* ^2^ = .20). | 16 |
| --- | --- | --- | --- | --- | --- | --- | --- | --- | --- | --- | --- |
| Hebl, King, and Lin (2004) | USA | 400 | 56% female,  44% male | 32.5% Caucasian,  23.3% African  American, 22.3% Asian American, 22% Hispanic | Unspecified | College students | Trying on a swimsuit vs. trying on a sweater | TST | GRE-Q | Participants in the swimsuit condition scored higher on SSO than participants in the sweater condition *(p* < .01). Participants in the swimsuit condition scored worse on a math test than participants in the sweater condition (*p* < .01). Neither gender nor race/ethnicity were significant moderators of this effect. | 14 |
| Kozak, Roberts and Patterson (2014) | USA | 80 | Female only | 63.8% Caucasian,  10% Hispanic, 6% Asian American, 3% African  American, 2% Native American/Pacific Islander | Unspecified | College students | Trying on a tank top vs. trying on a sweatshirt | TST | Raven's Progressive Matrices, GRE- Q | There was no main effect of shirt condition (tank top vs. sweatshirt) on SSO. However, in the tank top condition, participants sitting upright scored higher on SSO than participants in a slouched position (*p* = .009, *d* = .88). There were no main effects or interactions for shirt or posture on test performance. | 14 |
| Quinn, Kallen, Twenge, and Fredrickson (2006) | USA | 79 | Female only | 26.6% Asian  American, 25.3%  Caucasian, 25.3%  Hispanic, 22.8% African American | *M* = 21.30,  *SD*  unspecified | College students and community | Trying on a swimsuit vs. trying on a sweater | TST | Modified Stroop test | Participants in the swimsuit condition scored higher on SSO (*p* <  .01) and body shame (*p* < .01) than participants in the sweater condition. Participants in the swimsuit condition exhibited slower reaction times on a Stroop test (*p* < .05). | 12 |
| Tiggemann and Boundy (2008) | Australia | 96 | Female only | Unspecified | *M* = 19.71,  *SD* = 3.48 | College students | Entering an objectifying environment (i.e., a room with scales, mirrors, and magazine covers) vs. entering a non- objectifying environment; receiving an appearance compliment vs. receiving no appearance  compliment | TST | Nonsense Syllogisms Test, Cube Comparisons Test | There was no main effect of environment on SSO. However, participants high in CSO scored higher on SSO in the objectifying environment than participants low in CSO (*p* < .05). Participants who received an appearance compliment reported decreased negative affect (*p* < .01) but increased body shame (*p*  < .01). Participants high in CSO scored higher on body shame in the appearance compliment condition than participants low in CSO (*p* < .05). There was also a main effect of CSO on body shame such that participants who scored higher on CSO scored higher on body shame overall (*p* < .05). Test performance did not vary between conditions. | 13 |
